# Research Progress of Supramolecular Gels in the Field of Petroleum Engineering

**DOI:** 10.3390/gels11080661

**Published:** 2025-08-19

**Authors:** Liyao Dai, Jinsheng Sun, Kaihe Lv, Yingrui Bai, Jianlong Wang, Chaozheng Liu, Mei-Chun Li

**Affiliations:** 1 School of Petroleum Engineering, China University of Petroleum (East China), Qingdao 266580, China; b22020047@s.upc.edu.cn (L.D.); sunjsdri@cnpc.com.cn (J.S.); lkh54321@126.com (K.L.); smartbyron@163.com (Y.B.); 2 State Key Laboratory of Deep Oil and Gas, China University of Petroleum (East China), Qingdao 266580, China; 3 CNPC Engineering Technology R&D Company Limited, Beijing 102206, China; wjldr@cnpc.com.cn; 4 Co-Innovation Center of Efficient Processing and Utilization of Forest Resources, College of Materials Science and Engineering, Nanjing Forestry University, Nanjing 210037, China; lczwood@163.com; 5 Shandong Key Laboratory of Oil and Gas Field Chemistry, China University of Petroleum (East China), Qingdao 266580, China

**Keywords:** supramolecular gel, non-covalent bond, lost circulation control, fracturing fluids, profile control

## Abstract

Traditional petroleum engineering materials have problems such as single functionality and poor environmental adaptability in terms of lost circulation control and enhanced oil recovery. Supramolecular gels, with their dynamic reversible non-covalent network structure, demonstrate unique advantages in this regard. This paper classifies supramolecular gels into hydrogen bond type, metal coordination type, host–guest type, and electrostatic interaction type based on differences in crosslinking structures. It explains the construction principles and characteristics of each type of gel and analyses their application progress in petroleum engineering fields, such as lost circulation control in drilling, temporary plugging in fracturing, and profile control in enhanced oil recovery. It also discusses the advantages and disadvantages of different systems and future development directions. Research has shown that the molecular design strategy of supramolecular gels can effectively address technical challenges under complex conditions, offering new insights for oil and gas field development. Further optimization of their long-term stability and large-scale production technology is needed to advance their practical application.

## 1. Introduction

As a core component of the global energy system, petroleum not only occupies a key position in energy supply and industrial raw materials but also profoundly influences the global economic and political landscape. With the continuous growth in global demand for oil and gas resources and the gradual depletion of conventional oil and gas reserves, the strategic value of unconventional oil and gas resources (e.g., shale oil, tight oil, and coalbed methane) is becoming increasingly apparent. However, the development of unconventional oil and gas resources faces complex geological conditions (e.g., low permeability reservoirs, high temperature and pressure environments, heterogeneity), and traditional oil and gas development technologies are unable to efficiently address core issues such as lost circulation, reservoir modification, and recovery rate improvement. Existing petroleum engineering materials (e.g., rigid particle lost circulation materials, polymer gels, chemical oil displacement agents) have limitations such as single functionality, poor environmental adaptability, and non-degradability. There is an urgent need for new functional materials to break through technical bottlenecks.

Supramolecular gels, as smart materials constructed based on non-covalent interactions, provide a solution for the development of unconventional oil and gas resources. In the 1930s, Wolf in Germany coined the term “supramolecular” to describe the ordered systems formed by molecular association. In 1967, Pederson [1] first discovered that crown ether molecules can selectively bind alkali metal cations, revealing that the molecular aggregate form is a key factor influencing the selectivity of chemical reactions. Subsequently, Cram [2] proposed host–guest chemistry, and in 1978, Lehn [3,4,5] first proposed the complete concept of “supramolecular chemistry” by simulating the self-assembly structure of proteins. In the complex environment of petroleum engineering wells, water-soluble crosslinked polymer systems represented by polyacrylamide (PAM) [6,7,8], polyacrylic acid (PAA) [9], polyethylene glycol (PEG) [10], and polyvinyl alcohol (PVA) are widely used due to their simple preparation [11,12]. However, their insufficient stability under high-temperature (>80 °C) and high-mineralization (>100,000 mg/L) conditions limits their application in complex geological formations. Research shows that PAM exhibits significant viscosity decay and structural failure in high-temperature environments. While heavy metal ions or phenol-based crosslinking agents can improve temperature resistance, they pose environmental risks and economic challenges [13,14,15]. In contrast, preformed particle gels (PPGs) can avoid the uncertainty of crosslinking underground, but their expansion characteristics prior to injection make pumping difficult, and their mechanical properties are significantly reduced after water absorption, making them prone to losing their sealing effect [6,16]. Responsive gel systems (e.g., temperature, CO_2_, and pH-responsive systems) can achieve controlled gelation, but the trigger condition window is narrow [17]. For example, the state transition range of N-isopropylacrylamide-based systems is extremely small. The polymer network based on acrylamide and polyethyleneimine is overly dependent on CO_2_ stimulation response and responds slowly in heterogeneous formations [18,19]. Inorganic systems (e.g., cement and sodium silicate gel) have high temperature (150 °C) and long-term stability (>14 days), but their non-degradability means they lack dynamic regulation capabilities, making them more suitable for permanent formation sealing [20,21,22]. These inherent defects highlight the urgent need to develop new gel systems.

Addressing the technical challenges faced by existing materials in petroleum engineering, such as the dual failure of thermal stability and chemical stability, poor adaptability between static structures and dynamic environments, and imprecise response triggering in heterogeneous formations, supramolecular gels offer innovative solutions for engineering applications in complex formation conditions. This is due to their unique non-covalent bonding mechanisms, such as hydrogen bonding, hydrophobic interactions, and host–guest recognition. In terms of extreme temperature adaptability, supramolecular forces (e.g., hydrogen bonds and synergistic electrostatic interactions) enable the material to maintain structural stability across a wide temperature range of –20 to 120 °C. Hydrogen bonds and hydrophobic interactions enable the material to maintain an elastic modulus of 0.16 MPa under aging conditions at 135 °C [23]. Compared with materials such as PAM, supramolecular gels have better sealing capabilities for fractures and porous media [24,25]. In terms of tolerance to high-salt formations, the electrostatic interactions between sulfonic and amino groups can resist the compression double layer effect of salt ions, providing a new perspective for the application of polymers in ultra-high-salt reservoirs [26]. In terms of complex fluid migration, the dynamic hydrogen bond network endows the material with rapid recovery capabilities after shearing. Under high-temperature, high-shear conditions (200 °C 170 s^−1^), the material’s viscosity remains at 95 MPa·s. This makes it easy to pump while also enhancing the suspension capacity of proppants through a macro-thickening effect, thereby meeting the dual requirements of “low-friction pumping and high-viscosity sand transport” in fracturing operations [27,28]. In terms of improving recovery rates, object-recognizing supramolecular preformed gel particles (PPG) better address issues such as the irreversibility of curing materials, demonstrating a 10 higher sealing efficiency than traditional PPG in low-permeability rock cores of 0.05 mm [29,30]. Compared with traditional materials, supramolecular gel materials have significant advantages in terms of stability, responsiveness, and environmental compatibility, providing key material support for efficient lost circulation control, intelligent fracturing, and green oil displacement in deep oil and gas development. We have classified the reservoir conditions according to the following criteria:

Temperature: low temperature (<60 °C), medium temperature (60–120 °C), and high temperature (≥120 °C);

Salinity: based on total dissolved solids (TDS), low salinity (<35,000 mg/L or 3.5 wt%), medium salinity (35,000–100,000 mg/L or 3.5–10 wt%), and high salinity (>100,000 mg/L or >10 wt%);

Pressure: low pressure (<20 MPa), medium pressure (20–40 MPa), and high pressure (40–60 MPa);

Shear conditions: low shear rate (<0.01 s^−1^), medium shear rate (0.01–10 s^−1^), and high shear rate (>10 s^−1^).

This paper reviews the research progress of supramolecular gels, classifying them according to their crosslinking mechanisms into hydrogen bond type, metal coordination type, host–guest type, hydrophobic interaction type, and electrostatic interaction type, and elaborates on the characteristics and construction principles of each type of supramolecular gel. At the same time, the paper provides a detailed analysis of the research progress in the application of supramolecular gels in lost circulation control, temporary plugging in fracturing, and profile control in enhanced oil recovery. It explores the performance advantages of different supramolecular gel systems and their future development directions, providing a reference for further research and application of supramolecular gels in the field of petroleum engineering.

## 2. Classification of Supramolecular Gels

### 2.1. Hydrogen Bond

**Figure 1:**
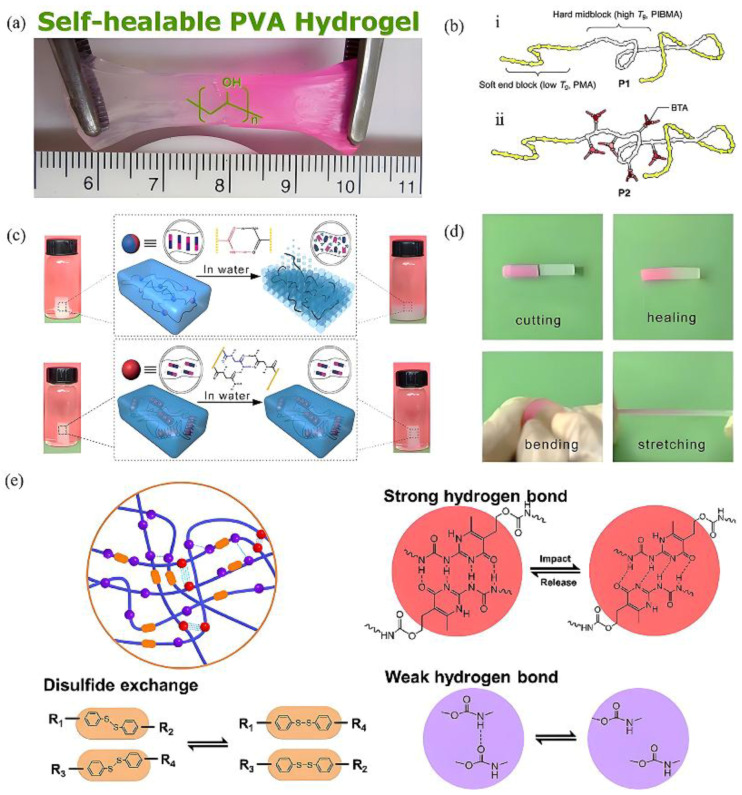
(**a**) The stretching process of PVA gel [33]; (**b**) (**i**)—schematic diagram without BTA, (**ii**)—schematic diagram with BTA in the middle chain segments [36]; (**c**) stabilization mechanisms of monoamide hydrogen bond gel (**top**) and bisamide hydrogen bond gel (**bottom**); (**d**) the self-healing, bending, and tensile properties of supramolecular gels [35]; (**e**) supramolecular gel based on quadruple hydrogen bond crosslinking and disulfide bond crosslinking [39]. Reprinted with permission from [33,35,36,39].

Hydrogen bonds, the most prevalent form of supramolecular interaction, were initially theorized by Moore and Winmill [31]. It has been established that hydrogen bonds typically form between hydrogen-containing molecules and atoms with higher electronegativity (e.g., O, F, and N). Following the formation of a polar covalent bond, the partial positive charge carried by the hydrogen atom is able to interact non-covalently with an electronegative atom in another molecule, thereby forming a hydrogen bond. Gel materials based on three-dimensional network structures formed by hydrogen bonds are one of the most widely used types of supramolecular hydrogels. Hassan et al. [32] conducted a systematic study of the gelation mechanism and structural stability of polyvinyl alcohol (PVA) supramolecular gels using the freeze–thaw method (Table 1). This study elucidated the formation mechanism of hydrogen bond-type supramolecular gels. Furthermore, Zhang et al. [33] discovered that PVA gels prepared by the freeze–thaw method can achieve self-repair at room temperature (Figure 1a). This phenomenon has been attributed to the presence of high concentrations of hydroxyl groups. This provides a theoretical basis for the study of self-healing properties. However, the strength and directionality of single hydrogen bonds is relatively weak, which makes it difficult to meet the requirements for the preparation of supramolecular gel materials. A substantial body of research has demonstrated that the formation of multiple hydrogen bonds can result in a substantial enhancement of a substance’s mechanical properties [34]. For instance, Dai et al. [35] introduced diamide groups into the glycine structure to synthesize the polymerizable monomer N-acryloylglycinamide (NAGA). The presence of diamide groups in the side chain of NAGA results in the formation of high-strength poly-N-acryl-glycinamide (PNAGA) supramolecular gels in water. In comparison with poly acrylamide (PAAm) with a single amide group, the double hydrogen bond structure of PNAGA gel was shown to be capable of effectively resisting the influence of water molecules, thereby significantly improving the stability of the gel network. Its elongation at break exceeded 1400%, and it exhibited high toughness and recoverable deformation capacity (Figure 1c,d). Furthermore, it has been demonstrated that the mechanical properties of the material can be further enhanced by the introduction of hydrogen bond motifs into the polymer intermediate segments. For instance, Hosono et al. [36] prepared a triblock copolymer with a middle block containing benzene-1,3,5-tricarboxamide (BTA) (Figure 1b). The amide bonds in the BTA molecules have been observed to form triple hydrogen bond structural units through hydrogen bonding interactions, thereby increasing the Young’s modulus of the material by 225% compared to materials devoid of BTA, reaching 360 MPa. Quadruple hydrogen bonds are another effective way to improve the mechanical properties of supramolecular gels. Ureido-pyrimidone (UPy) structures are often used to prepare high-performance supramolecular gels due to their ability to form quadruple hydrogen bonds. Sijbesma et al. [37] reported the thermodynamic control of the polymerization network in the UPy structure with four hydrogen bond binding sites, whose binding strength was significantly higher than that of the triple hydrogen bond structure. Based on this, Zhang et al. [38] prepared a dynamically responsive three-block supramolecular gel with poly (ethylene oxide) as the central block, combined with poly (N-isopropylacrylamide) (PNIPAm) and UPy groups. In circumstances where the temperature exceeded the lower critical solution temperature, the terminal segments underwent dehydration, resulting in the formation of micelles. These micelles served to create a hydrophobic environment conducive to the UPy groups, thereby establishing a three-dimensional physical gel network. This process was dynamically reversible, thereby endowing the gel with excellent self-healing and dynamic conversion capabilities. In order to enhance the strength of the gel further, Qin et al. [39] introduced quadruple hydrogen bonds, disulfide bonds, and nanosilica into polyurethane elastomers with a view to preparing a supermolecular gel with high mechanical strength (Figure 1e). The results showed that the formation of quadruple hydrogen bonds significantly increased the tensile strength of the gel (up to 3.951 MPa), and that the presence of multiple hydrogen bonds endowed the gel with excellent self-healing properties. Supramolecular gels formed by hydrogen bonds offer a combination of stability, flexibility, and self-healing properties, indicating their potential applications in petroleum engineering.

### 2.2. Metal Coordination

**Figure 2:**
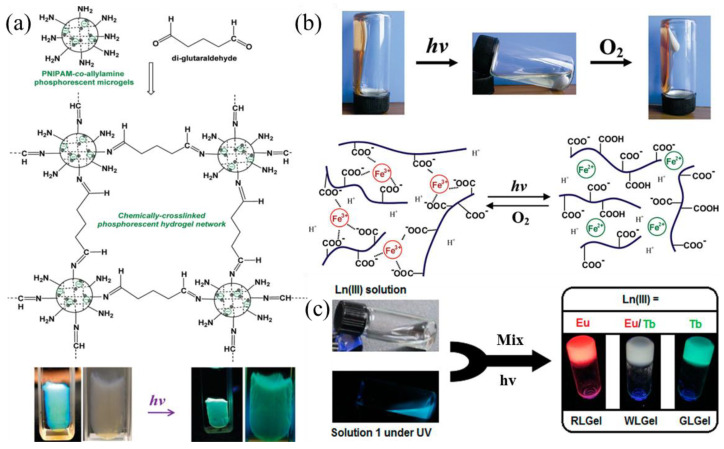
(**a**) Schematic diagram of the formation of hydrogel networks [40]; (**b**) schematic diagram of the dynamic conversion process and mechanism of gel–sol–gel [41]; (**c**) optical property changes based on coordination under ultraviolet light [42]. Reprinted with permission from [40,41,42].

Due to the unique properties of metals, research based on coordination interactions between metals and organic ligands has attracted increasing attention. Metal coordination bonds can be formed from various metal ions and organic ligands. These bonds have a clear directionality, and their bond energy is close to that of covalent bonds. These bonds are formed when two or more ligands interact with the empty orbitals of metal ions through their lone pairs of electrons. There are currently two main strategies for constructing such supramolecular gels. The first involves preparing gel factors containing metal ions and allowing them to interact to form gels. For instance, Marpu et al. incorporated gold (I) into a poly (N-isopropylacrylamide) (PNIPAM) polymer network, successfully producing phosphorescent microspheres that respond to stimuli. Changes in temperature, pH value, and ion concentration all significantly affected the luminescence intensity (Figure 2a) [40] (Table 2). Another method involves directly utilizing the coordination interaction between metal ions and ligands to form supramolecular gels. For example, Peng et al. [41] developed a redox-responsive gel–sol system based on the coordination interaction between trivalent iron ions (Fe^3+^) and carboxyl groups. In the system, Fe^3+^ formed coordination bonds with the oxygen atoms of the carboxyl group to create a gel network. When reduced by light, Fe^3+^ was converted into ferrous ions (Fe^2+^), which caused the gel to break down into a colorless solution (Figure 2b). If the solution came into contact with oxygen, the Fe^2+^ was re-oxidized to Fe^3+^, restoring the gel state. This process could be repeated multiple times. In addition, introducing metal ions can endow supramolecular gels with special functions. For instance, Chen et al. [42] developed luminescent gels based on terpyridyl-end-capped four-arm poly (ethylene glycol) polymer and lanthanide coordination, whose optical properties can be precisely controlled by adjusting the stoichiometry of the lanthanide metal ions (Figure 2c). Peng et al. [43] reported on metal–organic gels (MOG) formed from organic ligand 2, 6-Bis (2-benzimidazolyl) pyridine, and copper ions. This material exhibits excellent electrocatalytic performance thanks to its abundance of metal active sites and has a wide range of potential applications in electrochemistry. Due to the specificity of metal ions and the diversity of coordination chemistry, metal–ligand gel materials have great potential for use in a wide range of applications.

### 2.3. Host–Guest Interaction

**Figure 3:**
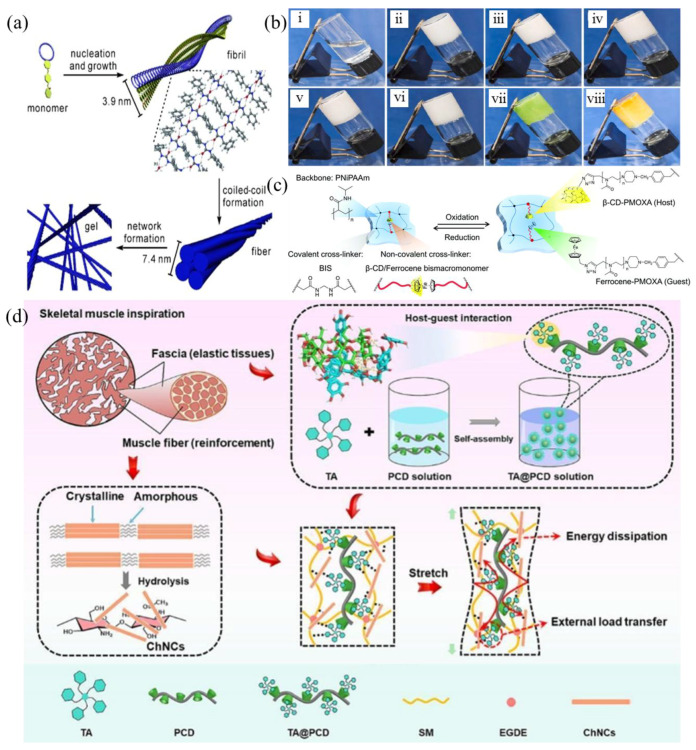
(**a**) Schematic diagram of gel layer-by-layer self-assembly [44]; (**b**) photos of gels formed under different conditions (**i**–**viii**) are, DMF, DMF/water, CaCl_2_, ZnCl_2_, BaCl_2_, MgSO_4_,CuCl_2_, and Fecl_3_ [45]; (**c**) schematic diagram of the molecular structure of the double crosslinked supramolecular gel system [46]; (**d**) schematic diagram of TA-PCD preparation [47]. Reprinted with permission from [44,45,46,47].

The interaction between host and guest molecules is crucial for the formation of supramolecular gels. This interaction is based on non-covalent bonding between the two types of molecule. Host molecules usually contain hydrophobic or hydrophilic cavities that can hold guest molecules. Common host molecules include crown ethers, cyclodextrins, calixarene, and cucurbiturils. Guest molecules tend to be organic compounds and metal ions. The development history of host molecules can be used to divide them into four representative generations. Crown ethers, as the first-generation host molecules, have relatively weak intermolecular forces and are typically not used alone to construct supramolecular gels. Qi et al. [44] incorporated crown ethers into fiber networks to create chiral gel agents capable of forming gels in various organic solvents. These agents exhibited stimulus-responsive properties through gel–sol transitions induced by K^+^ binding, pseudorotaxane formation, and anion binding (Figure 3a) (Table 3). Cyclodextrin (CD) is a second-generation host macromolecule whose structure is similar to that of a truncated cone. It features a hydrophobic cavity on the inside and exposed hydroxyl groups on the outside. CD can interact with guest units, such as ferrocene, adamantane, and azobenzene. CD can be obtained through starch hydrolysis. The most common types are α-, β, and γ-CD. Due to their water solubility, low cost, and good biocompatibility, they are widely used in the production of supramolecular gels. Ma et al. [45] discovered that metal ions can stabilize the tubular stacking structure of β-CD, thereby promoting the formation of supramolecular gels (Figure 3b). Liubimtsev et al. [46] developed a dynamic, dual-crosslinked, supramolecular gel system based on host–guest interactions between β-cyclodextrin (β-CD) and metal ions. This system comprised the following two networks: a covalent network formed by poly (N-isopropylacrylamide) and N,N-methylenebisacrylamide and a reversible crosslinked network formed by β-CD and ferrocene. This enabled the crosslinking points to be switched dynamically under redox conditions, demonstrating excellent repeatable cycling performance (Figure 3c). Given the cost-effectiveness of the host molecules (adamantane and azobenzene), Chen et al. [47] used naturally occurring plant polyphenol tannic acid to create a host–guest interaction with β-CD. They then added green reinforcing material in the form of chitin nanocrystals (ChNCs) to prepare a robust and resilient supramolecular gel (Figure 3d). Due to host–guest interactions and a crosslinked ChNC network, the gel material exhibited high wet and dry shear strengths of 1.25 and 2.57 MPa, respectively, as well as a gel toughness of 0.69 J. Furthermore, the material’s low gelation temperature indicates its potential for industrial applications. Calixarene are third-generation host molecules, consisting of phenol structural units linked by methylene groups. They are characterized by adjustable flexibility and ease of surface modification. However, unmodified calixarene have poor inclusion properties, so their derivatives are more important in the construction of supramolecular gels. Granata et al. [48] utilized micelle self-assembly technology to prepare supramolecular gels using choline and calixarene derivatives. These gels could be synthesized without the use of organic solvents or additives at a pH of 7.4, and they demonstrated remarkable self-healing properties. Cucurbituril (CB[n]) functions as the fourth-generation host molecule, composed of dimethyl bridge-linked glycerol urea. Among these, CB[6] and CB[7] have been observed to form 1:1 complexes with positively charged guest molecules, while CB[8] has been shown to form 1:2 host–guest complexes with guest molecules. The molecule’s highly symmetrical structure and excellent thermal stability have rendered it a highly sought-after novel host molecule. In this study, researchers utilized CB[8] as the primary component in the construction of supramolecular gels, incorporating 2-naphthol and methylviologen derivatives, the polymerizable guest molecule 1-benzyl-3-vinylimidazolium, and acrylamide [49]. It was evident that all of the gels displayed exceptional mechanical properties and self-healing properties. The unique properties of CB[8]uril have prompted researchers to explore various guest molecules, with a view to furthering the development of supramolecular gel materials.

### 2.4. Hydrophobic Interaction

**Figure 4:**
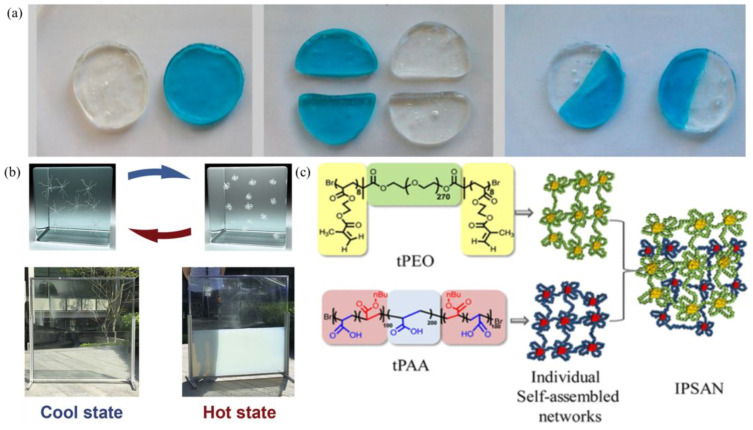
(**a**) Macroscopic images of two gel samples formed in 0.5 M NaCl and their self-healing process [50]; (**b**) the dynamic transition process of PNIPAm gel [52]; (**c**) schematic diagram of the interpenetrating network formed by amphiphilic triblock copolymers [53]. Reprinted with permission from [50,52,53].

Hydrophobic interaction-driven supramolecular gels represent a significant class of soft materials, whose formation mechanism primarily stems from the spontaneous aggregation behavior of hydrophobic groups in polymer chains in aqueous environments to reduce interfacial free energy. Hydrophobicity was first discovered in protein structure research, but with the increasing demand for material performance in specific application scenarios, traditional materials are no longer able to meet actual needs. In the course of their research, the scientists introduced hydrophobic functional groups into polymer chains, thereby achieving directed control of supramolecular gels. At low concentrations, hydrophilic segments form ring structures, while hydrophobic segments interact to form micelles. As the concentration increases, the micelles bridge each other through hydrophobic interactions, forming a three-dimensional supramolecular gel network. The process is subject to strict regulation by the ratio of hydrophilic and hydrophobic segments. For instance, Tuncaboylu et al. [50] utilized hydrophilic acrylamide and hydrophobic stearyl methacrylate and dococyl acrylate to execute a copolymerization reaction in a sodium dodecyl sulphate (SDS) micelle solution, thereby preparing a supramolecular gel (Table 4). The observed self-healing capabilities of the gel at room temperature, with elongation properties post-repair comparable to those of the original gel, were attributed to hydrophobic interactions (Figure 4a). This provided key theoretical support for the design of similar polymer gels. Block copolymers are another important approach for constructing hydrophobic supramolecular gels. Tsitsilianis et al. [51] synthesized a poly(styrene)-poly(tert-butyl acrylate)-poly(styrene) triblock amphiphilic copolymer, forms a micellar solution at a concentration of 0.2%, and when the concentration increases to 0.4%, the micelles are bridged by hydrophobic interactions to form a transient network structure, confirming the regulatory role of concentration in the gelation process. Concurrently, supramolecular gels comprising hydrophobic structures can be formed by thermoresponsive polymers, which effect gel–sol transitions by modulating temperature. Zhou et al. [52] prepared supramolecular gels based on poly (N-isopropylacrylamide) (PNIPAm). At environmental temperatures below the critical solution temperature, the amide groups in the segments were solubilized by water molecules, and the system remained transparent. When the temperature rose to a critical value, hydrophobic interactions increased, water molecules were released, particles contracted, and the system’s transmittance decreased, achieving a reversible gel–sol transition (Figure 4b). Klymenko et al. [53] prepared interpenetrating network gels with both UV- and pH-responsive crosslinking capabilities by mixing two solutions of triblock copolymers, thus expanding the environmental response dimensions of hydrophobic gels (Figure 4c). It is important to note that supramolecular gels that rely exclusively on hydrophobic interactions frequently exhibit limitations with regard to mechanical performance. Research has demonstrated that the mechanical strength of these materials is predominantly influenced by the temperature of the system and the concentration of polymers. In order to overcome this bottleneck, the following two improvement methods can be explored: firstly, enhancing the physical crosslinking density by increasing the polymer concentration; secondly, introducing synergistic mechanisms such as hydrogen bonds and host–guest interactions to construct a multi-scale composite supramolecular network.

### 2.5. Electrostatic Interaction

**Figure 5:**
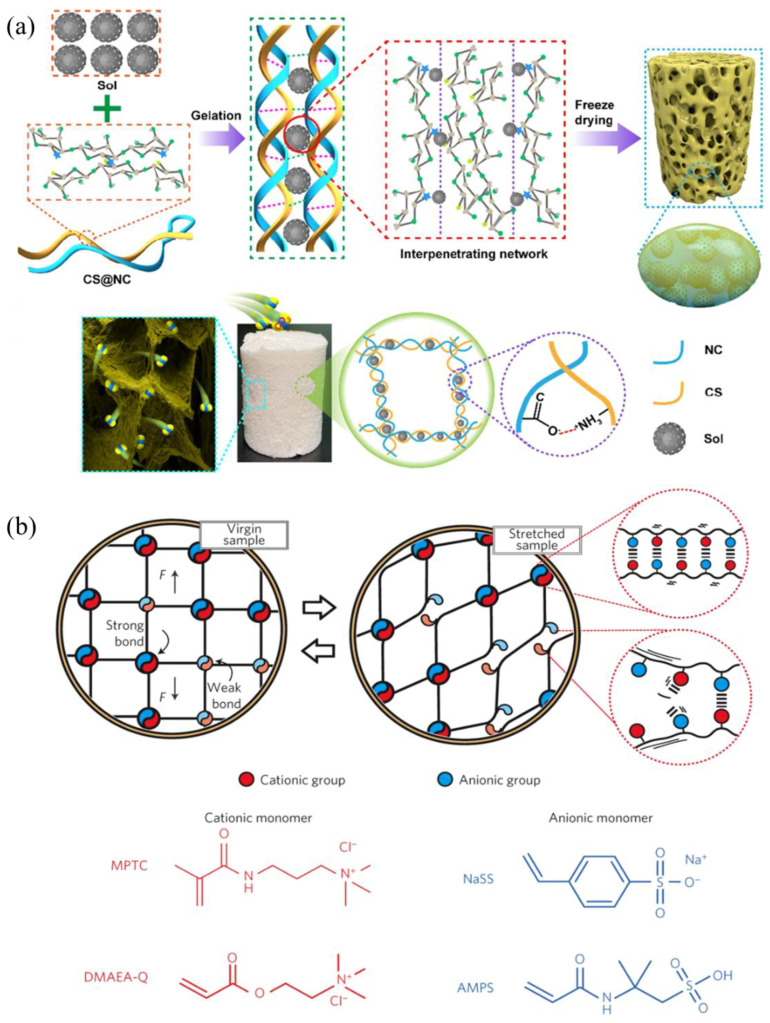
(**a**) Reactivity mechanism and preparation schematic diagram of supramolecular gel foam [55]; (**b**) diagram of bispodal polymer [56]. Reprinted with permission from [56,57].

Electrostatic interactions, as a typical type of non-covalent interaction, play an important role in the field of supramolecular materials. Electrostatic interactions arise from the Coulomb force generated by ions or groups with opposite charges through an electric field. Charged groups typically exhibit good water solubility and can form strong electrostatic interactions in water, which are non-directional and non-saturating. It is noteworthy that electrostatic interactions, as a conventional type of non-covalent interaction, exhibit comparatively elevated bond energies within such interaction forces. Specifically, their bond energies can range from 100 to 350 kJ/mol, a characteristic that enables electrostatic interactions to play a key role in many fields. In supramolecular gel systems, electrostatic interactions have been demonstrated to exhibit considerable advantages. Van Tomme et al. [54] were the first to develop a hydrogel system based on the self-assembly of oppositely charged groups. The design principle involves the mixing of positively and negatively charged dextran microspheres in equal volumes to form a physical crosslinked network through electrostatic interactions under neutral conditions, thereby achieving instant gelation (Table 5). This system exhibited unique thixotropic properties, with its physical crosslinked network capable of reversible breaking and reconstruction under stress. Zhang et al. [55] utilized electrostatic interactions between chitosan, nanocellulose, and silica sol to prepare supramolecular gel foams with stable CO_2_ adsorption performance (Figure 5a). The material exhibited sustained high adsorption performance across multiple cycles of use, thereby substantiating its structural stability. Moreover, this preparation strategy is consistent with the concept of sustainable development and provides new insights into the construction of multi-component gel systems. The ion monomer copolymerization strategy serves to expand the application range of electrostatic interactions. Sun et al. [56] employed electrostatic copolymerization of sodium p-styrenesulphonate with 3-(methacryloylamino)propyl-trimethylammonium chloride to prepare a supramolecular gel with 100% self-healing performance (Figure 5b). The system displays remarkable fatigue resistance through the reversible breaking and re-forming of non-covalent bonds, and its straightforward synthetic approach offers a novel method for the preparation of resilient hydrogels. Metal ion coordination, a distinct form of electrostatic interaction, has exhibited distinctive advantages in the field of gel reinforcement. Sun et al. [57] prepared a high-strength hydrogel with a tensile strength of 20 times its original length (fracture energy of 9000 J/m^2^) through ionic crosslinking between Ca^2+^ and carboxyl groups in polyacrylamide. In comparison with other non-covalent interactions, the supramolecular system constructed by electrostatic interactions has been shown to exhibit both a faster self-healing rate and a more stable network structure, attributable to the strong interaction between positive and negative ions. Electrostatic interactions demonstrate significant advantages in the construction of supramolecular gels through various modes of action (e.g., ionic self-assembly, multicomponent composites, ionic monomer copolymerization, and metal ion interactions), particularly in enhancing gel mechanical properties, imparting self-healing characteristics, and realizing functional applications.

## 3. The Application of Supramolecular Gels in the Oil Industry

### 3.1. Lost Circulation Control in Drilling

The core functions of drilling fluid, which is considered the “blood” of oil and gas drilling engineering, include cleaning the bottom hole, carrying cuttings, and balancing formation pressure. Concurrently, the filtration loss is regulated by the formation of a filter cake on the wellbore surface, thereby circumventing complex working conditions, such as lost circulation and wellbore instability. However, in the pore-fracture development strata, it is easy to cause complex accidents such as lost circulation, wellbore instability, and even blowout, resulting in significant economic losses. In view of this kind of leakage problem, the traditional inert plugging materials (e.g., walnut shell, calcium carbonate, fiber, resin, and cement) are difficult to realize the effective filling of the leakage section due to the weak interfacial force. In recent years, lost circulation materials based on supramolecular interactions have demonstrated significant advantages in the field of particles and gels due to their crosslinking self-healing properties.

**Figure 6:**
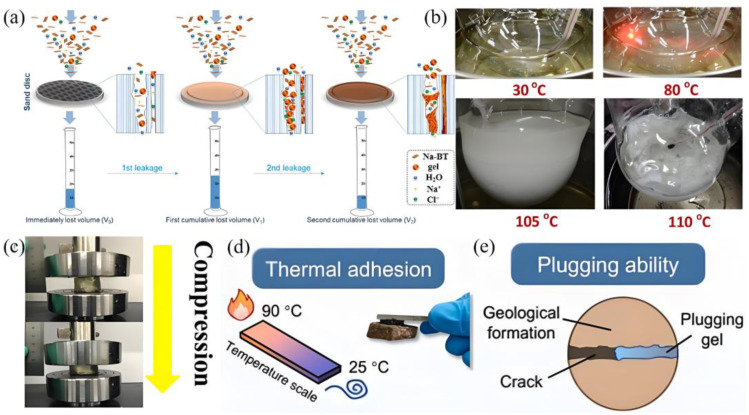
(**a**) The change process of gel particles in the plugging process [58]; (**b**) dynamic conversion of supramolecular gel materials at different temperatures [60]; (**c**) changes in the compression properties of supramolecular polymer gels [61]; (**d**) the adhesive properties of supramolecular gels; (**e**) schematic diagram of the sealing mechanism of supramolecular gels [63]. Reprinted with permission from [58,60,61,63].

Supramolecular gel particles dominated by hydrogen bonds show unique advantages for pore-microfracture leakage. Yang et al. [58] prepared self-healing amphoteric electrolyte gel particles using 4-Styrenesulfonic acid sodium salt (NaSS) and 3-methacrylamido-N,N,N-trimethylpropan-1-aminium chloride (MPTC). The particles interact with each other through hydrogen bonds, allowing them to deform and self-repair in water, thus compensating for the shortcomings of traditional gel particles and inert particles. This provides a useful reference for the field of lost circulation materials (Figure 6a and Table 6). However, for self-healing gel particles that primarily rely on hydrogen bonds, their rapid dynamic response characteristics may pose certain engineering risks. When the pumping flow rate is insufficient or the residence time in the wellbore is too long, the self-healing adhesion between the particles will occur in advance due to the recombination of hydrogen bonds, which is easily used to form a “sealing door” and will affect the normal pumping of drilling fluid and the subsequent plugging effect. In order to enhance the stability of the material, an appropriate amount of nano-silica was added to the above mixture. Non-covalent interactions such as strong hydrogen bonds and electrostatic interactions give microgels strong mechanical strength (1.03 MPa) and high temperature stability (150 °C) [59]. Furthermore, non-covalent interactions endow supramolecular gels with enhanced stimulus responsiveness, particularly their capacity for dynamic transformation in response to external stimuli (e.g., temperature and pH), which is of paramount importance for temporary materials. The host–guest chemistry based on β-CD provides a new strategy for dynamic plugging. The hydrophobic inner cavity of β-CD forms a three-dimensional network gel with organic guest molecules (e.g., long-chain alkanes) through hydrophobic interactions at elevated temperatures, thereby blocking leakage channels [60]. Upon attaining the temperature at which the gel-breaking threshold is reached, the host–guest complex structure is subject to disruption by Brownian motion. This results in the reversible transformation of the gel into a sol state, thereby facilitating subsequent unblocking operations (Figure 6b). This system addresses the drawbacks of traditional chemical gels, which are permanently sealed, through the temperature responsiveness of the interaction between the host and the guest, providing new ideas for the design of temporary plugging materials. Furthermore, the synergistic design of multiple non-covalent interactions has become a key strategy for improving the overall performance of gels.

The supramolecular polymer gel constructed from polyvinyl alcohol (PVA), acrylamide (AM), cellulose nanofibers (CNF), and laponite exhibited excellent thixotropic properties, facilitating pumping into formations; it exhibited high compressive strength due to the synergistic interaction of covalent bonds and multiple hydrogen bonds; and the gel demonstrated high compressive strength (0.69 MPa at 75% strain) and superior sealing performance (11 MPa) [61] (Figure 6c). In addition, considering the large amount of formation water in the actual reservoir, the elastic modulus of the supramolecular gel prepared using salt water (70,000 mg/L Na^+^ and 160,000 Ca^2+^) remained at 6369 Pa. The presence of salt water weakened some non-covalent interactions, but it was still possible to effectively seal the fractures in the reservoir. It has been demonstrated that the material exhibits high degradability under acidic conditions, thus meeting the requirements for reservoir protection. Meanwhile, adding nanomaterials to the above materials can further enhance the gel’s resistance to fluid intrusion [62]. After mixing with 50% drilling fluid, 100% salt water, and 10% heavy oil, the gel still maintained an elastic modulus of 5949 Pa (84 rad/s). This study provides a solution for the application of supramolecular gels in complex environmental conditions. Another type of gel system based on (3-acrylamidopropyl) trimethylammonium chloride (APTAC) maintains a bonding strength of 10 kPa within the temperature range of 25–90 °C through the synergistic effects of hydrogen bonding between polyphenol groups and cationic electrostatic interactions. In addition, after adding 5 × 10^4^ mg/L of inorganic salt, the adhesion performance was still maintained (6 kPa), addressing the issue of adhesive failure in traditional polymer gels (Figure 6d,e) [63]. In addition, thixotropic hydrogels constructed with AM, hydrophobic monomers (stearyl methylacrylate, SMA), kaolinite (KA), tannic acid (TA), etc., are endowed with chain segment flexibility through hydrogen bonds, electrostatic interactions, and hydrophobic interactions formed by physical crosslinking agents such as KA and TA, giving the gel-forming suspension good shear thinning and thixotropic properties, which are conducive to pumping to the bottom of the well and effectively remaining in the fracture [64]. This supramolecular gel holds significant potential for application in the petroleum and natural gas drilling industry. The performance of the material has been significantly improved due to the synergistic effect of the covalent network formed by the hydrophilic monomer AM and the hydrophobic monomer SMA and the reversible covalent bonds of the physical crosslinking agent. Adding 1% gel particles can effectively seal 60–80 mesh sand beds and remain stable under high-temperature and high-pressure conditions of 180 °C and 6 MPa. The cumulative leakage within 30 min was only 57 mL, demonstrating its excellent high-temperature sealing capability. In addition, for high-temperature formations, constructing a high-density flexible microgel through a combination of acylation reactions, phosphorylation, and hydrogen bonding is also an important approach [65]. Using a sealing device (wide lines of 19 cm in length with inner diameters of 4.0 and 2.5 mm were used to simulate fracture widths), the gel pressure reached up to 0.56 MPa after 24 h of aging at 180 °C. At the same time, the gel can also delay fluid leakage through deformation and compression, demonstrating its excellent dynamic sealing effect. This supramolecular gel holds significant potential for application in the petroleum and natural gas drilling industry.

The supramolecular gel system based on non-covalent interactions significantly improves the adaptability of lost circulation materials through the synergistic optimization of thixotropy, retention, dynamic responsiveness, and structural strength. The environmental friendliness and reservoir protection capabilities of such materials in high-temperature, high-salinity environments provide key technical support for the efficient development of unconventional oil and gas. By reasonably regulating the hydrogen bond density, the viscosity characteristics and salt resistance of the gel system can be improved. Furthermore, the synergistic effect of covalent crosslinking networks and non-covalent interactions gives the material excellent temperature resistance. However, in order to meet the demands of deep and ultra-deep drilling, future research needs to further improve the stability of materials under ultra-high temperature conditions.

**Table 6:** The application of supramolecular gels in drilling.

Regarding the Failure Mode	Material Type	Trigger Mechanism	Shear Behavior	Mechanical Properties	Key Performance	Laboratory/Field Application	Reference
The lack of sufficient interactions between material interfaces	Self-healing polyampholyte gel particles-H-bonding, cation-anion, and dipole-dipole interactions	Temperature (70–150 °C) and salinity (2–15% NaCl)	G′ increased to 14.6 MPa from 0.24 MPa (dimensions of 11.6 mm × 7.5 mm × 2 mm, frequency of 1 Hz, heating rate of 3 °C/min)	Compressive strength and tensile strength decreased from 7.3 to 1.9 MPa and 80.0–16.0 KPa, respectively, at 0–20% NaCl, 600% of strain after soaking in 20.0 wt % NaCl solution	Sand disk of 180 D: average porosity dropped from 9.86% to 1.06% (90 °C), 63.5% of the leakage volume reduction rate (LVRR); 63.4% LVRR at 15% NaCl and CaCl_2_ (90 °C); it can withstand a pressure of 6 MPa at 150 °C, 41.8% of LVRR	Laboratory stage	[58]
The lack of sufficient interactions between material interfaces	Zwitterionic polymer/nano-silica microgels-the strong hydrogen bonds, electrostatic interaction and cation-π	N/A	N/A	1.03 MPa of elongation, 217% of tensile deformation (120 mm/min of tensile speed)	Sand disks loss volume (5D): 17 mL at 80 °C, 29 mL at 150 °C, 37 mL at 7.5% NaCl (2% microgel after aging at 120 °C for 16 h)	Laboratory stage	[59]
The problem of the difficulty in degrading the temporary sealing materials	The reversible heat-set supramolecular gels-host–guest interactions and hydrogen bonding	Temperature(90 °C gel–110 °C sol)	G′ = 0.00001 Pa, 800 pa, and 100 pa at 30 °C, 95 °C, and 110 °C (0.01–10 Hz of frequency)	N/A	Fractured core test (0.5 mm): 6.8 MPa (105 °C)	Laboratory stage	[60]
The problem of the difficulty in degrading the temporary sealing materials	Cellulose nanofiber-reinforced supramolecular polymer gels- hydrogen bonding	N/A	G′ = ~20,000 Pa at 84 rad/s (10% of strain, 1–15 0.5–84 rad/s, 25 °C)	Tensile stress and elongation at break were 442 ± 35 kPa and 3212 ± 266%, 0.69 MPa of compressive strength at a fixed strain of 75% (50 mm/min tensile speed and a 20 mm/min compression speed, room temperature)	Sealing capacity (40–120 mesh, diameter of 45 mm and a length of 25 cm): 12.16 MPa (120 °C); Stability: the storage moduli adding brine was 6396 Pa (70,000 mg/L Na^+^ and 16,000 mg/L Ca^2+^)	Pilot field test	[61]
Insufficient adhesive capacity of the gel	Polydopamine embedded hydrogel-hydrogen bonding	N/A	N/A	65.3 kPa of the tensile strength, 570% of elongation; 9.9 kPa and 130% of tensile strength and elongation after adding 20 × 10^4^ mg/L (20 × 10 × 2 mm^3^, 100 mm/min of speed, room temperature)	Plugging test (1 mm): 7.6 MPa (90 °C); adhesion test: ~12 kPa, 8 kPa of PAM	Laboratory stages	[63]
Poor retention capability	Thixotropic polymer gel–hydrogen bonds, hydrophobic interactions, electrostatic interactions	N/A	G′ = 1000 Pa at 10 Hz (0.053 mm in thickness, 35 mm of diameter, room temperature)	N/A	Adhesion test: 0.62 ± 0.05 MPa; snad bed test (20–40 mesh): 27 mL (6 MPa and 150 °C); plugging test (8–16 mesh quartz sand): 5.3 MPa, 2.7 MPa of PAM (150 °C)	Laboratory stages	[64]

### 3.2. Temporary Plugging in Fracturing

**Figure 7:**
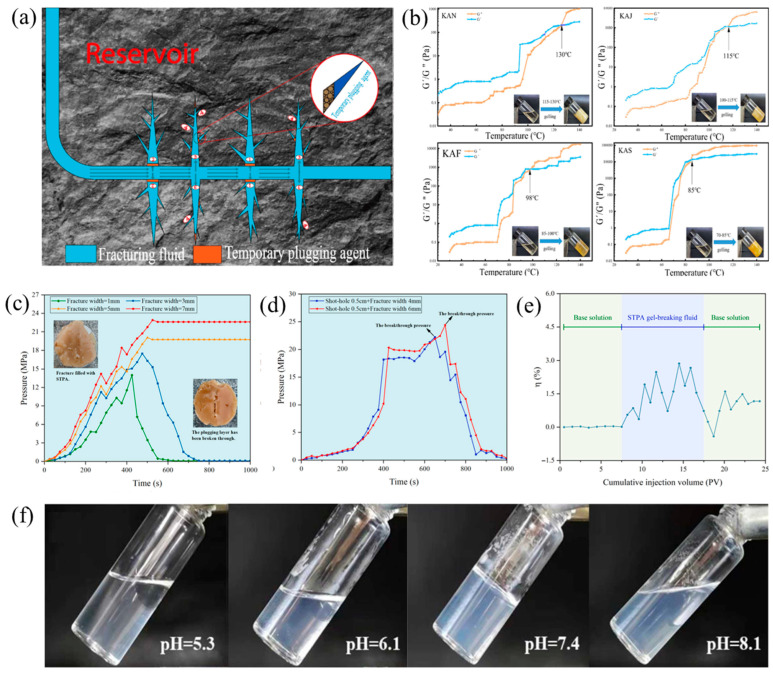
(**a**) Temporary plugging and sidetracking fracturing technology [66]; (**b**) the gelation behaviors of different systems: KAN does not contain any benzene derivatives; KAJ is added with toluene; KAF is added with phenol; KAS is added with benzoic acid [67]; (**c**) test of fracture sealing strength; (**d**) test of the maximum sealing strength of fractures and shot holes; (**e**) the curve of core loss test [69]; (**f**) the macroscopic states of supramolecular gels at different pH values [73]. Reprinted with permission from [66,67,69,73].

As conventional oil and gas exploration and development continue to advance, the difficulty of increasing reserves and production has increased, highlighting the strategic importance of unconventional oil and gas resources (e.g., tight oil, shale gas, and coalbed methane). Hydraulic fracturing technology plays a pivotal role in the development of such resources [66]. This technology has been demonstrated to effectively enhance reservoir transformation by artificially inducing the formation of a network of fractures, thereby increasing oil and gas production. However, during the fracturing process, fracturing fluid frequently flows preferentially into high-permeability areas or main fractures, making it difficult to effectively transform low-permeability areas and limiting the overall development effect of the reservoir. The technology of temporary plugging fracturing involves the pumping of a temporary plugging agent into the wellbore, with the objective of utilizing its sealing effect on existing fractures, thereby achieving dynamic diversion of the reservoir and expanding the volume of reservoir transformation (Figure 7a). At present, the most common fracturing plugging agents are those composed of fiber materials, plugging balls, and plugging particles. Despite the high plugging strength exhibited by these materials, their practical applications are constrained by several limitations. Firstly, it is difficult to effectively temporarily block deep fractures. Secondly, the degradation performance after sealing is not ideal, which can easily cause damage to the permeability of the reservoir. In addition, there are also risks such as gas channeling. In comparison, supramolecular gel fracturing plugging agents demonstrate significant advantages. In addition to their effectiveness in plugging fractures, their unique dynamic reversible properties facilitate subsequent unplugging operations, thus providing a new technical approach to unconventional oil and gas reservoir transformation.

Supramolecular gels, as a type of smart responsive material, have attracted considerable attention in the field of oilfield development due to their unique dynamic reversible properties. The material’s non-covalent bonding structure endows it with dynamic response characteristics to external stimuli (e.g., temperature, pH value), enabling reversible transformation between sol–gel–sol states. This controllable phase change provides new insights for temporary plugging technology in fracturing engineering. In response to changes in reservoir temperature gradients, a thermoresponsive supramolecular gel system constructed from cyclodextrin, benzene derivatives, and 1, 2-propylene glycol exhibits excellent temperature control characteristics [67]. It has been demonstrated that this system is capable of undergoing a “sol–gel” transformation during the process of heating (Table 7). Furthermore, by making adjustments to the type of benzene derivative, it was possible to precisely control the gelation temperature (70–130 °C). It can also be reversibly dissolved by secondary heating, meeting the dynamic requirements of “temporary blocking and unblocking” in fracturing operations (Figure 7b). The system has been shown to achieve a core sealing rate of over 85% and a degradation rate of over 98% at middle temperatures (>100 °C), thus meeting the engineering requirements for temporary blocking in low-permeability reservoir fracturing. In addition, cyclodextrin-based supramolecular gels have further expanded their applicable temperature range through composite with functional monomers [68]. However, this gel system primarily relies on weak intermolecular forces. It is evident that alterations in temperature have a substantial impact on intermolecular forces. It is evident that an increase in temperature results in a corresponding acceleration in the rate of bond breaking between molecules. Conversely, at lower temperatures, the gel structure is able to be sustained for a greater duration of time. The hydrogen bond-driven polysaccharide-based supramolecular gel system also exhibits excellent temperature-controlled response performance [69]. The pressure resistance of supramolecular gels was tested by continuously pumping the base fluid into the solution after it had gelled. The sealing strength of this supramolecular gel for 3 mm, 5 mm, and 7 mm fractures at 120 °C has been shown to exceed 12 MPa, and its pressure resistance in the “shot hole + fracture” model has been demonstrated to exceed 18 MPa (Figure 7c,d). This suggests that the gel displays excellent temporary sealing performance and effectively inhibits fluid flow to the original fracture. Concurrently, the gel caused only 1.2% damage to the permeability of the rock core and can be recovered through backflow after construction, causing minimal damage to the reservoir (Figure 7e). At the same time, supramolecular gels formed by liquid–solid transitions based on polyethylene glycol are also a viable technical approach [70,71]. pH-responsive supramolecular gels are also an important area of research [72]. By utilizing the hydrogen bonding between stearamide and different organic acids, a pH-responsive gel system can be constructed [73]. In this system, the protonation/deprotonation process of the tertiary amine and the dissociation behavior of maleic acid jointly regulate the formation and dissociation of the three-dimensional network structure. When the pH value of the system was below 7.4, the tertiary amine was completely protonated, and the system remained in a solution state. When the pH value exceeded 7.4, the degree of protonation of the tertiary amine decreased and the maleic acid completely dissociated, thereby enhancing intermolecular hydrogen bonding and promoting the formation of a three-dimensional network structure. The pKa of maleic acid (pKa_1_ = 1.9, pKa_2_ = 6.2) and amide (pKa = 9.4) determine their protonated states. When pH < 7.4, the protonation ratio of amides is >99% (Henderson–Hasselbalch equation), while only the first carboxyl group of maleic acid dissociates (pH < 5.3), and the system remains in solution. When pH > 7.4, maleic acid is completely dissociated (at pH 8.2, the second carboxyl group dissociation degree > 99%), the protonation ratio of the aminoamide decreases, and intermolecular hydrogen bonds form a three-dimensional network. During the plugging process, construction conditions with a pH of 7.0–8.0 are preferred to form “hydrogen bonds + moderate electrostatic” synergistic crosslinking, ensuring that the gel maintains its strength in the formation. During backflow treatment, conditions with a pH > 9 are used to achieve unblocking (Figure 7f). Core tests showed that the permeability recovery rate reached 97%. Compared with existing gels and fracturing plugging agents, this supramolecular gel plugging material demonstrated significant advantages in terms of pumping performance and reservoir damage control.

**Table 7:** The application of supramolecular gels in fracturing.

Regarding the Failure Mode	Material Type	Trigger Mechanism	Shear Behavior	Mechanical Properties	Key Performance	Laboratory/Field Application	Reference
Narrow gel transition temperature	β-cyclodextrin/benzene derivatives gel (KAN without adding any benzene derivates, KAJ adding toluene, KAF adding phenol and KAS adding benzoic acid)—host–guest interactions	Temperature (gelling at 70–130 °C), degradation time (1.2, 1.7, 2.6 and 3.2 h for KAS, KAF, KAJ, KAN)	N/A	N/A	Degradation rate: 98, 97.5, 97.6, 97.7% for KAS, KAF, KAJ, KAN; Plugging test: 5.6 MPa (~45 × 10^−3^ μm^2^), 6.9 MPa (~40 × 10^−3^ μm^2^), 7.4 MPa (~37 × 10^−3^ μm^2^) and 7.6 MPa (~36.5 × 10^−3^ μm^2^) for KAN, KAJ, KAF and KAS (KAN gelling at 130 °C, KAJ gelling at 115 °C, KAS gelling at 98 °C, KAF gelling at 85 °C)	Laboratory stages	[67]
Conventional gels are difficult to remove blockages	Phase change fracturing—β-CD-aliphatic alcohols and fatty acids	Gelatinizing temperature (83–122 °C)	N/A	N/A	Plugging test: 8.45 MPa (50 mm of core length, 0.5 mm of fracture width)	Laboratory stages	[68]
Poor adaptability, insufficient pressure-bearing capacity, and low efficiency of returning to the ground	Low-damage temperature-controlled phase change temporary plugging agent—hydrogen bonds	Gel formation time (10–40 min at 60–80 °C), Gel-breaking time (100–420 min at 120–160 °C)	20,000 Pa at 1 Hz (0.1 of shear stress, 80 °C)	N/A	Plugging performance tests: the fractures of 1 mm, 3 mm, 5 mm, and 7 mm all have a width greater than 14.2 MPa; the shot-hole of 0.5 mm size and 4 mm/6 mm sizes are all greater than 18 MPa (120 °C), damage performance: 1.2%.	Field application	[69]
Poor adaptability and low efficiency of returning to the ground	Thermoresponsive in situ-generated proppant based on liquid solid transition of a supramolecular self-propping fracturing fluids	Temperature (clear liquid at 30 °C, solid at 60, 90, and 120 °C)	G″ was always higher than G′ at 30 °C, (the stress and frequency were set as 1 Pa and 0.01–10 Hz)	N/A	Dynamic leak off tests: 0.0007 of matrix, 0.3 of fractures	Laboratory stages	[70]
Break-up in presence of slats and high temperature	Supramolecular assembly of maleic acid and an amino–amide charge interaction (2 wt%)	pH (4–10), 10.2 mPa·s at pH = 4, 2500 mPa·s at pH = 8.5	Viscosity: no total break-up or degradation at 5 wt% salt	N/A	52 kJ/mol supramolecular solution, polyacrylamide (~16 kJ/mol)	Laboratory stages	[71]
Low values of polymer injectivity and pumping efficiencies	Responsive Amphiphilic systems as displacement fluids—long-chain amino–amide and maleic acid	pH (3.7 × 10^4^ mPa·s at pH = 4, 4.5 × 10^5^ mPa·s at pH = 8)	N/A	N/A	2 wt% of adaptable amphiphile/maleic acid into water increased the viscosity of water by a factor of 4.5 × 10^5^	Laboratory stages	[72]
The gel breaking time and degree of gel breaking are still difficult to control	Quaternary ammonium-based supramolecular gel-hydrogen bonds	pH (solution at pH < 7.4, gel at pH = 7.4, sol at pH > 8.1) and temperatures	N/A	N/A	Viscosity (mPa·s): 1.63 × 10^5^, 4.35 × 10^5^, 5.02 × 10^5^, 6.12 × 10^5^ of SDA (azelaic acid derived TPA), SDT (tartaric acid derived TPA), SDC (citric acid derived TPA), SDM (maleic acid derived TPA); plugging rate (%): 29.05, 68.73, 74.95, and 90.43 of SDA, SDT, SDC, and SDM. (70 °C)	Laboratory stages	[73]

The supramolecular gel system constructed based on host–guest interactions and non-covalent bonds such as hydrogen bonds provides an innovative solution for fracturing temporary plugging technology due to its dynamic reversible properties. Cyclodextrin-based supramolecular gels exhibit significant thermoresponsive properties through host–guest interactions (β-cyclodextrin), and their phase transition temperature can be regulated by the structure of the guest molecule, enabling a reversible “sol–gel–sol” transition during heating. In addition, the pH-responsive system formed by stearamide and organic acids through a dynamic hydrogen bond network is also an effective way to construct pH-responsive gels. Nevertheless, in practical applications, this type of material still faces many challenges. Firstly, in comparison with conventional chemical temporary plugging agents, supramolecular gel systems triggered by temperature and pH value require dynamic regulation under specific conditions, and subsequent operations must be precisely controlled, resulting in high technical barriers. Furthermore, this type of material has poor adaptability in complex environments (e.g., high temperatures and high mineralization). It is important to note that this material is still in the laboratory research stage and requires further development to achieve technical maturity. In response to the above challenges, subsequent research needs to develop a multi-stimulus response coordination system to achieve dynamic responses of materials under diverse stimulus conditions. Furthermore, through the utilization of molecular structure design and composite modification technology, the temperature and salt resistance of supramolecular gels is enhanced, thereby improving the adaptability and stability of the material in complex environments. These breakthroughs will significantly enhance the engineering applicability of supramolecular gel plugging technology, promoting its transition from laboratory research to field application.

### 3.3. Profile Control in Enhanced Oil Recovery

**Figure 8:**
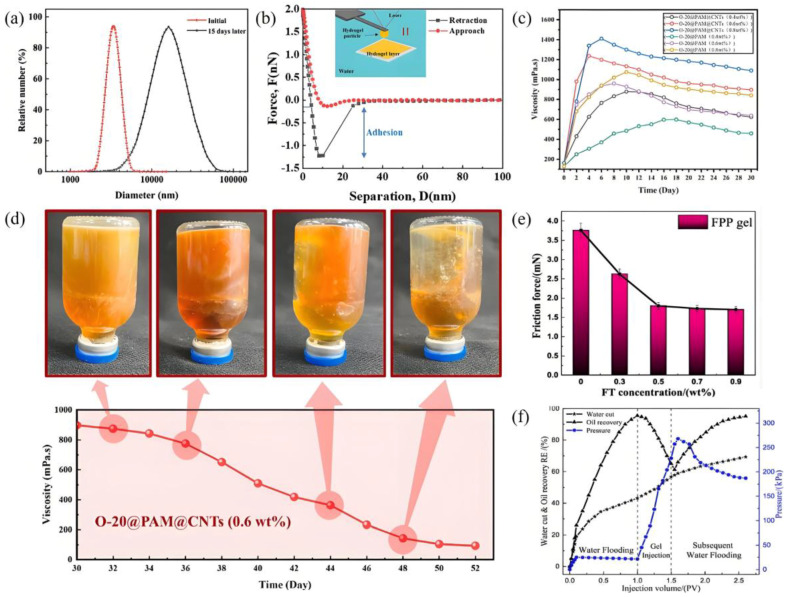
(**a**) The particle size distribution after 15 days of aging; (**b**) the adhesion force curve between the gel particles and the gel surface [75]; (**c**) the viscosity changes in the gel under different concentrations of crosslinking agents; (**d**) the change in the viscosity of the gel over time [76]; (**e**) gel self-lubrication performance relationship curve; (**f**) curves of the oil displacement experimental data [78]. Reprinted with permission from [75,76,78].

In the middle and late stages of oilfield development, oil wells generally face the technical problems of low recovery and high water content. In order to improve the efficiency of crude oil recovery, profile control technology is often used to transform the reservoir. The utilization of conventional profile control agents (e.g., granular materials, polymers, and foams) is encumbered by an inherent incompatibility between migration depth and plugging strength. In recent years, supramolecular gels based on non-covalent interactions have demonstrated considerable potential for application in profile control, owing to their distinct performance advantages. In order to enhance the interfacial interactions between gel particles, Gao et al. [74] constructed a supramolecular system based on phenol-formaldehyde resin, which utilizes the synergistic action of hydrogen bonds on the particle surface to efficiently block micron-sized pores. This provides a theoretical basis for the design of micro- and nano-scale profile control modifiers. It has been demonstrated through further studies that combining hydrogen bonding with π-π stacking interactions has the potential to significantly enhance the strength of interactions between gel particles. A supramolecular gel system that self-grows was constructed using catechol-functionalized partially hydrolyzed polyacrylamide and phenolic resin as the matrix [75]. After aging for 15 days in a reservoir environment (80 °C, 0.5 mol/L NaCl), the median particle size increased from 3.5 μm to 18 μm (Figure 8a and Table 8). Concurrently, the mean adhesive force between particles within the system attained 1.21 ± 0.04 nN (Figure 8b). By increasing the pore flow resistance, the blocking effect was enhanced, providing a new solution for deep profile control in heterogeneous reservoirs.

By regulating the number of physical crosslinking points between non-covalent bonds and polymer segments, gel systems with adjustable strength can be prepared to meet the profile adjustment requirements of reservoirs with different permeability. A gel system constructed using phenol and hexamethylenetetramine as crosslinking agents enables the gradient control of viscosity by adjusting the concentration of the crosslinking agents [76]. At low shear rates (0.1 rad/s), the gel can establish a static structure, and as the shear rate increases (0.1–100 rad/s), weak gels exhibit shear thinning behavior. At a crosslinking agent concentration of 0.6 wt%, the gel exhibited peak viscosities of 879 mPa s, 1237 mPa s, and 1190 mPa s at 90 °C, 110 °C, and 130 °C, respectively, indicating its ability to withstand high-temperature conditions. The gel demonstrated excellent stability, with a maximum viscosity of 1400 mPa·s and a maintenance viscosity of 907 mPa·s after 30 days. The formation permeability decreased from 313 mD to 27.3 mD after plugging, effectively improving the permeability distribution characteristics of heterogeneous reservoirs (Figure 8c,d). However, the performance of this gel in high-salinity environments (i.e., TDS > 100,000 ppm) has not yet been systematically verified through experimental testing, and its key properties, such as crosslinking stability and rheological characteristics, have not yet been experimentally validated. The presence of non-covalent interactions, such as hydrogen bonds, contributes to the maintenance of supramolecular gels’ mechanical properties, while also exhibiting thixotropy [77]. This property enables them to penetrate highly permeable reservoirs and achieve effective sealing, thereby enhancing deep profile control effects. Bai et al. [78] constructed a self-lubricating supramolecular hydrogel with shear-responsive properties by integrating N-fluorenylmethoxycarbonyl-l-tryptophan, polyacrylamide, and sodium polyacrylate. Unlike PAM-PAA gels, which maintain a constant storage modulus (G′) during shear cycling, supramolecular gels form ultra-low friction with solid surfaces within a certain shear cycling range (within 18 cycles). However, as shear cycling increases further, their friction and strength increase significantly (Figure 8e). This characteristic facilitates gel migration in formation channels and effectively seals high-permeability reservoirs. In addition, compared with PAM-PAA gel, the plugging rate, water flooding volume sweep efficiency, and oil recovery of supramolecular gel increased by 83.1%, 155.4%, and 34%, respectively (Figure 8f). It is important to note that high-temperature reservoir environments and long-term CO_2_ storage may induce acidic conditions, which can lead to deterioration in the performance of traditional profile control materials [79]. To address this issue, Luo et al. [80] developed a copolymer system that responds to both temperature and CO_2_. Under weakly acidic conditions, the responsive groups of this material protonate and self-assemble into a supramolecular network driven by non-covalent interactions. In comparison with conventional profile control technologies, this system can increase oil recovery rates by approximately 20%. While maintaining high sealing efficiency, it also has multiple environmental response characteristics, providing an important reference for the development of intelligent profile control materials. Furthermore, supramolecular organic gelling agents based on different alkyl chain structures (e.g., hydrogen bonds, π-π stacking, and van der Waals forces) exhibit rapid oil phase selective gelling capabilities, providing a new direction for selective plugging during profile control processes [81].

**Table 8:** The application of supramolecular gels in enhanced oil recovery.

Regarding the Failure Mode	Material Type	Trigger Mechanism	Shear Behavior	Mechanical Properties	Key Performance	Laboratory/Field Application	Reference
Migration distance and plugging strength	Self-growing hydrogel particles—hydrogen bonds	N/A	N/A	N/A	Median size of hydrogel particles: increased from 3.5 to 18.0 μm (aging for 15 days at 80 °C and 0.5 M NaCl solution)	Laboratory stages	[75]
Durability in harsh reservoir conditions	Hyperbranched, nanowire-prepared weak gels-hydrogen bonding and electrostatic interactions	N/A	0.8 wt% of crosslinker, 5637 mPa·s at 0.1 rad/s, G′ > G″ at frequency range (110 °C, 1–100 rad/s)	N/A	Core plugging test: permeability reduction from 313 mD to 9.9 mD, yielding a resistance factor of 31.3; (110 °C, 48 h, 217, 501 of TDS), oil recovery rate: 89.9% (110 °C, 217, 501 of TDS)	Laboratory stages	[76]
Environmental pollution problems	Urea-containing supramolecular polymer gel–hydrogen bonding	N/A	G′ > G″ at 0.1–100 Hz (1 mm of thickness, 17.5 mm of the radius, room temperature)	N/A	Pressure-bearing properties: 250 N (8.75% monomer gel) and 500 N (17.5% polymer gel) (150 °C)	Laboratory stages	[77]
Some deficiencies of gel in-depth profile control	Self-lubricating supramolecular hydrogel/π-π stacking, hydrogen bond	Shear rate	Gel–sol transition frequency of FPP-0.5 displayed a first stable and then significant increase trend with shear cycle rise (20–60 Hz at frequency, 25 °C)	N/A	Core flooding experiments (30 cm × 4.5 cm × 4.5 cm of size): 86.6 of the plugging rate, 83.5 of water flooding volumetric sweep efficiency, 71.3 of oil recovery (80 °C).	Laboratory stages	[78]
Gas channeling and mobility control	CO_2_-responsive and smart mobility control agent	CO_2_	33,000 mPa·s at 0.01 1/s (35 mm of d, 25 °C)	N/A	Plugging performance: 171 kPa, 99.2% of plugging efficiency (50 °C, 10,985 mg/L salinity)	Laboratory stages	[79]
Acidic and thermal hydrolysis of acrylamide/polyacrylamide	Smart polymer	CO_2_ and temperatures (solution in 25 °C, gel in 90 °C)	N/A	N/A	Core flooding test: increase 21–22% total oil recovery than the conventional a	Laboratory stages	[80]
Environmental problem	Alkyl bicarbamates supramolecular organogelators–H bonding, π-π stacking and van der Waals interactions	N/A	N/A	N/A	Oil removal rates are always higher than 95% and the oil retention rates can be close to 100%	Laboratory stages	[81]

Supramolecular gels constructed based on non-covalent interactions (e.g., hydrogen bonds, π-π, and van der Waals forces) possess self-assembly and self-responsive properties, rendering them promising candidates to address the shortcomings of existing profile control materials. Supramolecular polymer gels based on electrostatic interactions exhibit excellent thixotropic properties, and their dynamically reversible ionic crosslinked networks can significantly improve the permeability control effect of heterogeneous reservoirs. They can simultaneously meet the requirements of deep migration and high-strength sealing, thereby achieving stepwise profile control. However, the application of supramolecular gel materials in profile control is currently mostly in the laboratory research stage, mainly due to cost factors, with few field application studies. Furthermore, under harsh geological conditions, the crosslinking strength of supramolecular gels with single non-covalent crosslinks is relatively weak, which poses a significant challenge in meeting durability requirements. In response to the aforementioned issues, future research should focus on the following areas:

(1) Through molecular structure design, non-covalent interactions (e.g., hydrophobic association, hydrogen bonding, electrostatic interactions) are combined with covalent crosslinking to construct a composite crosslinked network, thereby enhancing the long-term stability of supramolecular gels.
(2) The incorporation of solid fillers (e.g., SiO_2_, CaCO_3_, asphalt nanoparticles) into supramolecular gel systems enhances material strength and reduces production costs through the synergistic effect of non-covalent interactions between the fillers.
(3) The development of a gel system capable of coordinating with multivalent metal ions (e.g., Ca^2+^ and Mg^2+^) in formation water is essential, as is the enhancement of the material’s mechanical properties through the coordination crosslinking effect of metal ions. This adaptation to complex reservoir environments is crucial.

### 3.4. Field Applications

In the field of petroleum engineering, the field application and pilot testing of supramolecular gels have achieved a series of phased results. Typical application examples in the field of lost circulation control are as follows: In the treatment of fracture lost circulation in the 1st well area of the Junggar Basin (lost circulation depth 3000 m, drilling fluid density 1.26 g/cm^3^) and in response to the technical bottleneck of conventional lost circulation materials being unable to stem leaks, the Bohai drilling engineering company has developed supermolecular gel particles with a particle size of 100–2000 μm that can swell during drilling [82]. This material combined elasticity and deformability. A formula of 6% comprehensive lost circulation material + 25% fruit shells + 1.5% supramolecular gel lost circulation material was used on site. After pumping in 40 m^3^ of lost circulation material, it was circulated and left to settle for 6–8 h, successfully restoring drilling. The lost circulation operation was successful, and this technology was repeatedly applied in three subsequent wells, including Well 205. Furthermore, with respect to the fracture lost circulation (rate of 20–30 m^3^/h) that occurred in the middle wells of the Bohai oilfield, neither bridge plugging materials nor cement materials could achieve the desired results [83]. China Oilfield services limited synthesized the gel polymer Gelseal, underpinned by the principles of supramolecular theory. The company employed a “gel + cement” synergistic lost circulation control technology, with the gel material isolated the formation fluid from the wellbore material, thereby providing a critical time window for the cement slurry to remain and solidify, thus achieving effective sealing of the loss channel. With regard to the implementation of supramolecular gel technology in the domain of enhanced oil recovery, its engineering value in the development of complex reservoirs has been demonstrated. In the Bohai Oilfield (1300 and 1600 m of depth, 65 °C of reservoir temperature, and 6071 mg/L of salinity), a hydrophobic associative polyacrylamide (HMHPAMS)-enhanced oil recovery system was employed [84]. Following the cumulative injection of 48,000 tons, there was an observed increase in crude oil production of 2.748 × 10^6^ m^3^. This represents a 5.1% improvement in recovery rate when compared to traditional partially hydrolyzed polyacrylamide (HPAM). Addressing the low production challenges of medium- to low-permeability gas wells in the East China Sea (formation pressure coefficient 1.20–1.42), Hu et al. [65] developed a high-temperature (180 °C) curing solid-free flexible microgel plug (SFMP), which was configured as a 1.5 g/cm^3^ saltwater plugging slurry and used to seal the lower formation. The instantaneous production rate of the gas well increased to 3900 m^3^/h, with a daily production rate of 9.36 × 10^4^ m^3^. Addressing the issue of production decline caused by oil pressure drop in the later stages of gas well development, Li et al. [69] pumped 16 m^3^ of supramolecular plugging agent into a gas well. Compared with the lost circulation material used previously, the pumping pressure increased by 12.08 MPa, and the gas production increased by 15,100 m^3^/d, showing a significant effect on production enhancement. These applications not only validate the applicability of supramolecular materials in the development of complex oil and gas reservoirs but also provide a reliable technical path for improving the efficiency of oil and gas field development.

Field trials have strongly confirmed the feasibility and effectiveness of supramolecular gels in petroleum engineering applications. With increasingly stringent environmental requirements, the petroleum engineering field has set higher standards for the degradation performance of materials, and supramolecular gel materials based on non-covalent interactions have demonstrated significant advantages. It has been demonstrated that the pH response characteristics have the capacity to reduce viscosity in an acidic environment, thereby enhancing pumping efficiency. Following injection into the reservoir, contact with an alkaline environment has been demonstrated to restore high viscosity, thereby reducing migration rates. This dynamic response capability provides a favorable path for reducing environmental pollution [85]. Despite the efficacy of high-strength polymer gels and cement-based materials in temporary occlusion, the subsequent unplugging process (e.g., high-concentration acidification) carries significant environmental and economic implications that cannot be disregarded. Such operations serve not only to increase the duration of oil well operations but also to exacerbate environmental problems such as soil acidification and groundwater pollution [86,87,88]. In addition, some polysaccharides added to supramolecular materials can be continuously obtained from plants [89], with raw material costs ranging from USD 800 to 1500/ton. However, materials such as HPAM not only cost as much as USD 2000 to 5000/ton but also have neurotoxicity, which can have adverse effects on the human body [90]. The components of supramolecular gels (e.g., stearic acid and xanthan gum) can be derived from natural materials, combining cost-effectiveness with environmental sustainability. Stearic acid (C18 saturated fatty acid), with a price range of USD 500–1000/ton, can be extracted from plant oils such as cocoa butter and shea butter. Xanthan gum, a natural polymer, is priced at USD 1500–4000/ton and can be produced through the fermentation of sugars, such as glucose and sucrose [72,85,90]. This hybrid supramolecular gel raw material is more economical than synthetic polymers and can also reduce the use of non-biodegradable synthetic polymers. Concurrently, the biodegradability of natural components has the potential to mitigate long-term ecological risks, including soil and groundwater pollution. The majority of supramolecular gels are costly and complex to synthesize, which hinders their deployment in field applications. In addition, there are still many challenges to be overcome before supramolecular gels can be used on a large scale in industry. Some supramolecular gels containing different alkyl chains can only form organic gels in solvents [91]. Polymer preparation must be carried out under neutral conditions and under nitrogen protection to maintain performance, resulting in a highly complex synthesis process [92]. Currently, supramolecular gel materials are mostly in the laboratory stage and cannot meet the demands of large-scale continuous industrial production. Therefore, developing synthesis processes and equipment suitable for industrial-scale manufacturing, improving production efficiency and product quality stability, is the key to promoting their large-scale application.

In the practical application of supramolecular gels, the trade-off between cost and performance is a key issue. When utilizing natural polymers, such as xanthan gum, cellulose, and sodium alginate, as gel materials, it should be noted that, despite the evident benefits of straightforward synthesis processes and minimal raw material costs, their performance is susceptible to environmental factors, including temperature and salinity. Supramolecular gels incorporating non-covalent interactions such as metal coordination can enhance stability and mechanical strength through crosslinking networks, but they come with increased costs and potential environmental risks [45]. In order to improve the performance of supramolecular gels while reducing costs, introducing more inorganic materials is an effective strategy. Lapointe can be introduced to improve the retention performance of the solution [93]. Coal fly ash, as an industrial waste residue, is widely available and inexpensive [94]. Adding it to gel systems can effectively enhance the mechanical properties of the gel. SiO_2_ and clay can increase the mechanical strength and toughness of the gel, improving its overall performance [95]. Through judicious selection and integration of these inorganic materials, the performance of the material can be ensured while maintaining cost efficiency [96,97]. From the perspective of sustainable development, actively exploring green synthesis is an inevitable trend for future development. Bio-based monomers are derived from renewable resources such as vegetable oils and starch. This approach has two main benefits. Firstly, it reduces dependence on traditional petroleum-based raw materials, and secondly, it lowers production costs. Furthermore, it minimizes the environmental impact of gel preparation processes. After application in petroleum engineering, controllable degradation of the gel can be achieved through enzyme-based designs [98]. For difficult-to-degrade systems, it is necessary to develop recycling and reuse technologies or explore new applications such as shape memory functions to reduce environmental risks and improve resource recycling efficiency.

## 4. Conclusions

The present study systematically analyses the potential application of supramolecular gels in the field of petroleum engineering and draws the following conclusions:

(1) Supramolecular gels constructed based on non-covalent interactions (hydrogen bonds, metal coordination, host–guest recognition, hydrophobic interactions, and electrostatic interactions) combine high structural strength, dynamic reversibility, and functional designability, effectively addressing key technical challenges, such as lost circulation, temporary plugging during fracturing, and enhanced oil recovery in complex geological conditions.
(2) Different crosslinking mechanisms confer differentiated properties on gel materials. Hydrogen bonding and metal coordination significantly enhance mechanical strength, while host–guest interactions enable dynamic reversible regulation. Hydrophobic interactions ensure stability in high-temperature, high-salt environments, and electrostatic interactions provide self-healing and functionalization properties.
(3) In the current study, such as the lost circulation, the host–guest gels achieve dynamic fracture sealing through temperature-triggered hydrophobic crosslinking, while hydrogen bond gels enhance fracture interface bonding strength through high adhesion (10 kPa). In fracturing fluid, the host–guest supramolecular gel formed by cyclodextrin and benzene derivatives exhibits excellent temperature control properties, meeting the dynamic requirements of “sol–gel–sol” in fracturing operations. In profile control, hydrogen bonds and π-π bonds enhance the interparticle interaction forces, with an average interparticle adhesion force of 1.21 ± 0.04 nN. The synergistic effects of hydrogen bonds, π-π bonds, and van der Waals forces provide more options for profile control.

It is recommended that future research efforts concentrate on the development of supramolecular gel systems that exhibit elevated levels of toughness and stability. This should be achieved through the synergistic design of covalent–non-covalent hybrid crosslinking mechanisms and multiple non-covalent interactions. At the same time, biosynthetic technology and green chemical processes should be utilized to reduce raw material costs and establish an environmentally friendly system. Combining molecular dynamics simulation and reservoir numerical simulation, the mechanism of action of supramolecular gels in complex environments should be analyzed in depth to support the efficient development of deep and unconventional oil and gas resources.

## Figures and Tables

**Table 1:** Hydrogen bond-based supramolecular gels.

Composition	Preparation Method	Testing Condition	Performance	Application	Reference
Hydrogen bonds/PVA (15%)	8 h freezing at −20 °C and 4 h thawing at +25 °C, 12 h gelation	Swelling studies were conducted in deionized water at 25 °C	19% after 26 Day	Biomedical applications	[32]
Hydrogen bonds/PVA (35%)	Freezing/thawing method, 13 h gelation	Room temperature, tensile rate of 1 mm/s	1.0 × 10^5^ Pa (fracture stress)	biomedical applications	[33]
Hydrogen bonds/PVA/tannic acid (TA)	Freezing/thawing method, 6 h gelation	Strain rate is 50 mm/min, gels shape (30 mm × 5 mm × 1 mm), room temperature	Tensile strength and elongation achieve 4.9 MPa and 950%.	Intelligent devices, energy storage, tissue engineering	[34]
Hydrogen bonds/N-acryloyl glycinamide (NAGA)	Photoinitiated aqueous-phase polymerization	Gels with thickness of 0.5 mm, 50 mm in length and 6 mm in width, the extension of 50 mm/min, room temperature	Tensile strength and elongation achieve 1.1 MPa and 1400%. (room temperature)	Biomedical field	[35]
Hydrogen bonds/ABA triblock copolymers containing benzene-1,3,5-tricarboxamide	At 40 °C for 12 h	20 °C	Young’s modulus: 360 MPa, fracture strain 203%. (room temperature)	Engineering thermoplastics	[36]
Hydrogen bonds/four cooperative hydrogen bonds	Units of 2-ureido–4-pyrimidone	N/A	N/A	N/A	[37]
Hydrogen bonds/a central poly(ethylene oxide) block and terminal poly(N-isopropylacrylamide) (PNIPAm) block with ureido pyrimidinone (UPy) (10 wt%)	Heated to 37 °C for hydrogelation	40 mm of diameter, 1 rad/s of frequency, 10% of polymer concentration (37 °C)	γ = 0.5%, G′ = 3000 Pa.	Biomedical applications	[38]
Hydrogen bonds/supramolecular polyurethane	Mixing at 55 °C for 3 h	Mechanical and self-healing tests: dimension of 10 × 5 × 0.6 mm, room temperature	Mechanical and self-healing tests: dimension of 10 × 5 × 0.6 mm, room temperature	Protective materials	[39]

**Table 2:** Metal coordination-based supramolecular gels.

Composition	Preparation Method	Testing Condition	Performance	Application	Reference
Au (I)-ligand	A water-soluble phosphorescent Au(I) and poly(N-isopropylacrylamide) (PNIPAM), 4 °C for chemical crosslinking	N/A	Temperature-dependent photoluminescence enhancement of the hybrid microgel upon heating from room temperature (RT) to 37 °C, only PNIPAM-co-allylamine microgels at acidic pH showed efficient loading of the Au phosphor	Various biological and environmental applications	[40]
Fe-ligand	Aqueous PAA solution containing an Fe (III)-citrate complex, the gel–sol transition in the PAA + Fe (III)-citrate aqueous system switched by photoreduction and oxidation	N/A	N/A	N/A	[41]
Ln-ligand	lanthanide metal–ligand coordination complexes via a terpyridyl-end-capped four-arm poly(ethylene glycol) polymer	Room temperature	Variety of reversible stimuli-responsive properties (mechano-, vapo-, thermo-, and chemochromism) of both sol–gel systems	Smart coatings	[42]
Copper (II)-ligand	Organic ligand 2,6-Bis (2-benzimidazolyl) pyridine and copper (II)	N/A	N/A	Electrochemistry material	[43]

**Table 3:** Host–guest interaction-based supramolecular gels.

Composition	Preparation Method	Testing Condition	Performance	Application	Reference
The crown ether represents a molecular recognition unit	Benzo-21-crown-7 ether	25 °C	Three different chemical stimuli that induce gel–sol transitions: K binding, pseudorotaxane formation, and anion binding, 10 kPa of G′	N/A	[44]
β-Cyclodextrin and metal ions	The CD was mixed with deionized water containing metal ions and then ultrasonicated for 15 min before being left to stand	0.105 of thickness, 35 mm diameter (room temperature)	Yield stress values of GelCaCl_2_, GelZnCl_2_, GelBaCl_2_, GelMgSO_4_, GelCuCl_2_ and GelFeCl_3_ are 466 Pa, 469 Pa, 573 Pa, 833 Pa, 936 Pa and 460 Pa, respectively	Intelligent materials, drug delivery	[45]
β-cyclodextrin and ferrocene	Poly (N-isopropylacrylamide) (PNiPAAm) backbone crosslinked with N,N′-methylenebis (acrylamide) (BIS) as a permanent crosslinker, and β-CD/ferrocene host–guest complexes as reversible crosslinking points	5 mm of thick slice (room temperature)	15% of swelling degree	Microfluidics and diagnostics	[46]
Chitin nanocrystal (ChNCs), soybean meal (SM), and β-cyclodextrin	Tannin acid (TA)-functionalized poly-β-cyclodextrin (PCD) (TA@PCD) and chitin nanocrystals (ChNCs) into the SM matrix	Bonding performance: at 25 °C for 10 min to test the wet strength, 25 mm × 25 mm of bonding area, the adhesive endured 500 g and was maintained for 80 days (25 °C)	2.57 and 1.25 MPa of dry and wet shear strength (dry for 3 h at 120 °C), 0.69 J of adhesive, the cost of SM/TA@PCD/ChNCs-2 adhesive was approximately 2290 RMB (or 322 US dollars)/ton, which was comparable to that of the commercial urea-formaldehyde resins (around 2100 RMB (or 295 US dollars)/ton), thus showing great promise for wood adhesive applications	Biomass adhesives	[47]
Choline-calix arene derivative and curcumin	Choline-calix arene derivative and curcumin mixture was sonicated for 15 min, then stirred (500 rpm) at 37 °C	40 mm of diameter, frequency sweep at 2% strain with varying angular frequency (1–25 rad/s), 25 °C	No significant changes in G′ and G″ within the applied range of angular frequency (1–10 rad/s) (25 °C)	Drug delivery	[48]
Cucurbit[8]uril	Poly (ethylene glycol) (PEG) and cis-1,4-poly(isoprene) (PI) were chosen as the polymers	N/A	N/A	Dynamic functional materials	[49]

**Table 4:** Hydrophobic interaction-based supramolecular gels.

Composition	Preparation Method	Testing Condition	Performance	Application	Reference
Strong hydrophobic associations between stearyl methacrylate (C18) and dococyl acrylate (C22)	C18, C22, acrylamide in a micellar solution of sodium dodecyl sulfate (SDS)	4 mm diameter × 50 mm length, 50 mm/min of speed, 25 °C	The original and healed gel samples broke at elongation ratios of 3600 ± 630 and 3580 ± 520%, respectively, indicating a healing efficiency of about 100%, 12 ± 1 kPa of tensile strength	Biological materials	[50]
Polystyrene (PS) end-capped polyelectrolytes	Modification of polystyrene-poly (tert-butyl acrylate)-polystyrene triblock copolymers synthesized via anionic polymerization	50 mm of diameter, 1% concentration, 25 °C	1000 Pa at 100 rad/s	Paints, cosmetics, drilling fluids	[51]
Poly (N-isopropylacrylamide)	N-isopropylacrylamide, N,N-methylenebis, and N,N,N,N-tetramethylethylenediamine mixing at 600 rpm for 30 min, then the solution was left stirring for at least 24 h	N/A	N/A	Energy-saving buildings	[52]
Amphiphilic BAB triblock copolymers	Mixing the same volumes of the P (nBA50%-stat-AA50%)-b-PAA-b-P(nBA50%-stat-AA50%) (tPAA) solution and the poly(2-methacryloyloxyethyl acrylate)-b-poly(ethylene oxide)-b-poly(2-methacryloyloxyethyl acrylate) (tPEO) solution	0.103 mm of thickness, 25 mm of diameter	1000 Pa at 10 rad/s (after UV-irradiation, 20 °C)	N/A	[53]

**Table 5:** Electrostatic interaction-based supramolecular gels.

Composition	Preparation Method	Testing Condition	Performance	Application	Reference
Oppositely charged crosslinked dextran microspheres	The dispersions (Hydroxyethyl methacrylate-derivatized dextran and methacrylic acid) were stored at 4 °C for 2 h	500 μm of thickness, 20 mm of diameter	6500 Pa of G′ with a solid content of 25%. (20 °C and 1 Hz)	Drug delivery	[54]
Oxidized nanocellulose (NC) and Chitosan (CS)	NC, CS, and silica sol mix thoroughly for 2 min, then the samples were freeze-dried at 60 °C for 12 h	At room temperature (25 °C) for 5 h	The N_2_ adsorption capacity was 241.18 cm^3^/g STP, and the CO_2_ adsorption capacity was 23 mL/g	Inhibitor material	[55]
Polyampholytes	Cationic monomers: 3-(methacryloylamino)propyl-trimethylammonium chloride (MPTC), N,N-dimethylamino ethylacrylate (DMAEA-Q); anionic monomers: sodium p-styrenesulphonate (NaSS), 2-acrylamido-2-methylpropanesulphonic acid (AMPS). Polyampholyte hydrogels were synthesized using the one-step random copolymerization of an anionic monomer and a cationic monomer	12 mm (L) × 2 mm (d) × 2–3 mm (w), 100 mm/min of rate, room temperature	1.8 MPa and 750% of fracture stress σb and strain ɛb values, 4000 J/m^2^ of the tearing energy, reached ~30% after 1 h healing	Smart structural materials	[56]
Ca^2+^ and carboxyl groups	Hydrogels by mixing two types of crosslinked polymer (onically crosslinked alginate, and covalently crosslinked polyacrylamide)	75.0 × 5.0 × 3.0 mm^3^ of size, 2 mm/min of rate, room temperature	29 kPa of elastic modulus, ~2000% of strain rate, ~9000 J/m^2^	Tissue engineering	[57]

## Data Availability

No new data were created or analyzed in this study. The original contributions presented in this study are included in the article. Further inquiries can be directed to the corresponding author.
